# Cannabidiol Is a Potential Inhibitor of Ferroptosis in Human Articular Chondrocytes

**DOI:** 10.1111/jcmm.70592

**Published:** 2025-06-27

**Authors:** A. Wipplinger, D. Bekric, C. Ablinger, M. Kittl, C. Mayr, M. Ritter, M. Winklmayr, M. Jakab

**Affiliations:** ^1^ Center of Physiology, Pathophysiology and Biophysics, Institute of Physiology and Pathophysiology Paracelsus Medical University Salzburg Austria; ^2^ Ludwig Boltzmann Institute for Arthritis and Rehabilitation Salzburg Austria; ^3^ Institute of Pharmacy Paracelsus Medical University Salzburg Austria; ^4^ Department of Internal Medicine I Paracelsus Medical University/Salzburger Landeskliniken (SALK) Salzburg Austria; ^5^ Gastein Research Institute Paracelsus Medical University Salzburg Austria

**Keywords:** CBD, chondrocytes, ferroptosis, OA, osteoarthritis, viability

## Abstract

The present study investigates the effects of cannabidiol (CBD), the major non‐psychoactive compound of *Cannabis sativa* L. extracts, on ferroptotic cell death in human articular chondrocytes. Exposure to known ferroptosis inducers RSL3, erastin and its analogue IKE, FINO2 and FIN56 led to a varying extent of reduced cell viability in two chondrocyte cell lines (in C‐28/I2, T/C‐28/A2) and primary chondrocytes, suggesting different sensitivity and defence mechanisms towards the respective substances. The cytotoxic effects were aggravated by additional exposure to iron and inhibited by the specific ferroptosis inhibitor ferrostatin‐1 (Fer‐1), proving the occurrence of ferroptosis. Strikingly, co‐treatment of ferroptosis inducers with CBD clearly restored cell viability in a dose‐dependent manner (10 nM to 1 μM CBD) in both cell lines and primary chondrocytes. Moreover, CBD restored the activity of GPX4, a major anti‐oxidative enzyme, to varying degrees when combined with IKE or RSL3. Increasing evidence has emerged for an important role of iron dyshomeostasis and ferroptosis in the onset and progression of various orthopaedic diseases, including osteoarthritis. Therefore, the here demonstrated and previously unreported cytoprotective and anti‐oxidative effects of CBD in the context of ferroptosis have highly promising therapeutic implications.

**Abbreviations**
AB/AM: antibiotic‐antimycotic
CBD: cannabidiol
DMSO: dimethyl sulfoxide
DPBS: Dulbecco's phosphate‐buffered saline
ECM: extracellular matrix
Era: erastin
Erastin: eradicator of RAS and ST‐expressing cells
FAC: ferric ammonium citrate
FBS: foetal bovine serum
Fer‐1: ferrostatin‐1
FIN: ferroptosis inducer
FINO2: 1,2‐dioxolane
GPX4: glutathione peroxidase 4
GSH: glutathione
HRP: horseradish peroxidase
IKE: imidazole ketone erastin
Nec‐1: necrostatin‐1
OA: osteoarthritis
resazurin: 7‐hydroxy‐3H‐phenoxazin‐3‐one‐10‐oxide
ROS: reactive oxygen species
RSL3: RAS‐selective lethal 3
SD: standard deviation
SDS: sodium dodecyl sulfate
STS: staurosporine
Z‐VAD‐FMK: carbobenzoxy‐valyl‐alanyl‐aspartyl‐[O‐methyl]‐fluoromethylketone

## 1 Introduction

In 2012, Dixon et al. [1] described ferroptosis as a new form of iron‐dependent regulated cell death. Excessive accumulation of membrane lipid peroxides to toxic levels, which disturbs the composition, structure and dynamics of lipid membranes and their constituents, is one of the hallmark characteristics of ferroptosis [2, 3, 4]. Lipid peroxides are generated from polyunsaturated fatty acids by hydroxyl and peroxyl radicals produced in the Fenton reaction [4]. Ferroptosis is functionally, biochemically and morphologically distinct from other forms of regulated cell death, such as apoptosis or necroptosis, and is not prevented by their specific inhibitors. However, it can be suppressed by ferrostatins, which inhibit lipid peroxidation, or by iron chelators like deferoxamine [5, 6].

Cells utilise various anti‐ferroptotic mechanisms that can be targeted to induce ferroptosis. Four classes of ferroptosis inducers (FINs) are frequently used in research. Class I FINs like erastin or its analogue IKE inhibit the cystine/glutamate antiporter system x_c_^−^ (SLC7A11), which mediates cellular cystine uptake. Cystine is reduced to cysteine, which is utilised for the synthesis of the major endogenous antioxidant glutathione (GSH). Class II FINs like RSL3 directly inhibit glutathione peroxidase 4 (GPX4), which acts as a lipid repair enzyme by reducing lipid peroxides to lipid alcohols. Class III FINs (e.g., FIN56) cause GPX4 depletion, whereas class IV FINs like FINO2 oxidise unstable Fe^2+^ and indirectly inactivate GPX4 [7, 8, 9].

Ferroptosis is linked to pathological conditions including cancer, neurodegeneration, stroke, kidney injury and infection [10]. In recent years, the role of iron and impaired iron homeostasis in the pathogenesis of age‐related diseases has been recognised [11] and an association between ferroptosis and orthopaedic diseases could be shown [12]. Osteoarthritis (OA), a leading cause of chronic disability in the ageing population, is characterised by progressive cartilage damage, synovial inflammation, subchondral bone alterations and osteophyte formation [13]. A growing body of clinical evidence indicates a correlation between iron dyshomeostasis and cartilage damage. Structural joint damage is observed in patients with hereditary haemochromatosis and haemophilia, potentially caused by systemic iron overload or local iron accumulation from joint bleeds [14]. Moreover, increased serum ferritin levels positively correlate with cartilage damage in OA patients, and synovial fluid iron levels are higher in OA patients than in healthy subjects, correlating with OA severity [15, 16, 17]. A genome‐wide study in African Americans revealed a significant correlation between iron transport pathways and knee OA [18]. Additionally, it was found that antioxidant system activity and lipid peroxidation influence OA severity [19].

The association between ferroptosis and OA is supported by studies in animal models and in vitro (reviewed in Cao et al. [20] and Zhang et al. [19]). Disruption of iron homeostasis by inflammatory cytokines, mechanical overload or FINs was found to induce cartilage degeneration in animal models and to cause decreased matrix production and up‐regulation of matrix‐degrading enzymes in cultured chondrocytes. Conversely, beneficial effects of ferroptosis inhibitors, iron chelators or lipophilic antioxidants have been reported.

Cannabidiol (CBD), a non‐psychoactive component of *Cannabis sativa* L. extracts, directly and indirectly modulates redox functions and has anti‐oxidative as well as oxidative properties, depending on cell type, dose, exposure time and context [21]. We previously demonstrated pro‐apoptotic effects of CBD in micromolar concentrations in the immortalised chondrocyte cell line C‐28/I2 and primary human articular chondrocytes [22].

In the present study, we investigate whether CBD in the sub‐micromolar range affects ferroptosis in the immortalised chondrocyte cell lines C‐28/I2 and T/C‐28/A2. Furthermore, key results obtained from the cell lines were also verified in primary human chondrocytes. Our results strongly suggest that CBD dose‐dependently counteracts ferroptotic cell death in chondrocytes.

## 2 Materials and Methods

### 2.1 Reagents

Erastin and IKE were obtained from Selleck Chemicals (Cologne, Germany). FIN56, ferrostatin‐1, necrostatin‐1, RSL3 and Z‐VAD‐FMK were from MedChemExpress (Monmouth Junction, NJ, USA) and FINO2 from Cayman Chemical (Ann Arbor, MI, USA). Ferric ammonium citrate and resazurin were from Merck Sigma‐Aldrich (Darmstadt, Germany). Cannabidiol was purchased from Abcam (Cambridge, UK). CBD was dissolved in ethanol, and all others in DMSO. Rising doses of the respective reagents were tested in viability assays in advance to choose adequate concentrations for the following experiments. Likewise, solvents were tested at the highest used concentrations prior to experiments to exclude solvent effects.

### 2.2 Cell Culture

C‐28/I2 and T/C‐28/A2 cells originating from human rib cartilage [23, 24] were cultured in DMEM/F‐12 medium (Gibco, Thermo Fisher Scientific, Darmstadt, Germany) supplemented with 5% foetal bovine serum (FBS‐Maximus; Catus Biotech, Tutzing, Germany) and AB/AM solution (100 U/mL Penicillin, 0.1 mg/mL Streptomycin, 0.25 μg/mL Amphotericin‐B; Merck Sigma‐Aldrich) in a humidified atmosphere at 37°C and 5% CO_2_.

Primary chondrocytes were isolated from finger cartilage obtained from body donors within 24 h post‐mortem. Donors were part of a body donation programme of the University Center of Anatomy and Cell Biology, Paracelsus Medical University Salzburg in accordance with ethical guidelines and legal requirements. Donors had given prior written consent for the use of their tissues for research purposes. After extraction, the tissue was transferred into DPBS (PAN‐Biotech, Aidenbach, Germany) and centrifuged. DPBS was discarded, and cartilage was transferred to sterile DMEM/F‐12 medium (Gibco) supplemented with 5% FBS (FBS‐Maximus; Catus Biotech), 1% AB/AM solution (100 U/mL Penicillin, 0.1 mg/mL Streptomycin, 0.25 μg/mL Amphotericin‐B; Merck Sigma‐Aldrich), collagenase type‐2 (2 mg/mL; Gibco) and gentamicin (50 μg/mL, 1:1000; Merck Sigma‐Aldrich). The tissue was shaken overnight at 110 rpm and 37°C and then dispersed using a 40‐μm Falcon cell strainer. After centrifugation, cells were cultured in T‐25 flasks (Sarstedt, Nümbrecht, Germany) in fresh DMEM/F12 medium.

### 2.3 Cell Viability

#### 2.3.1 Resazurin Viability Test

Ten thousand cells per well were seeded into transparent 96‐well microplates (CytoOne; Starlab, Hamburg, Germany) and grown overnight. After 24 h, the medium was replaced by medium without FBS (−FBS) and grown for a further 24 h before treatment. Cells were then incubated with the different substances in medium −FBS at concentrations as indicated in the individual experiments. Samples were incubated at 37°C for 24 h, and then the supernatants were replaced by 100 μL medium −FBS containing 0.5 mM resazurin. After 1 h, supernatants were transferred to a new 96‐well plate and either measured immediately or stored at −20°C until measurement. Fluorescence was measured in a Spark multimode reader (Tecan, Grödig, Austria) in triplicates per experimental condition. Blank well values (medium only) were subtracted, and cell viability was related to untreated control cells.

#### 2.3.2 CellTiter‐Glo Luminescent Cell Viability Assay

Cells were seeded as for resazurin assays. After starvation, the cells were incubated with the different substances in 100 μL medium −FBS for 24 h. After 24 h, 100 μL of CellTiter‐Glo substrate (Promega, Walldorf, Germany; Cat.‐No. G7570) was added, and plates were transferred on a shaker for 2 min. After 10 min at room temperature, supernatants were transferred to a white 96‐well plate (Greiner Bio‐One, Kremsmünster, Austria) and luminescence was detected on a Spark multimode reader (Tecan). Different experimental conditions were measured in triplicates.

### 2.4 Microscopy

Ten thousand cells per well were seeded into transparent 96‐well plates. After 24 h, medium was changed to −FBS. After a further 24 h, the indicated substances diluted in 100 μL medium −FBS were added, and the plate was transferred into a Spark multimode reader (Tecan) in a humidity cassette for long‐term observation at 37°C and 5% CO_2_, with pictures taken at 10× magnification every hour over 24 h.

### 2.5 Caspase 3/7 Activity

Cells were seeded for resazurin assays. Twenty‐four hours post‐treatment in 100 μL total volume of medium −FBS per well, 100 μL of Caspase‐Glo 3/7 assay substrate (Promega; Cat.‐No. G8090) was added and the assay was performed according to the manufacturer's instructions. Luminescence was measured on a Spark multimode reader (Tecan). Different experimental conditions were measured in triplicates.

### 2.6 Intracellular Iron Content

2.6 × 10^6^ cells were seeded in 100‐mm culture dishes (Sarstedt) and grown overnight. Before treatment with substances as indicated, cells were starved in medium −FBS for 24 h. Then cells were washed with DPBS (PAN‐Biotech) and harvested with trypsin/EDTA (Merck Sigma‐Aldrich). Before harvesting, pictures of cells were taken at 20× magnification with a Lumenera Infinity 8‐9 camera (Teledyne Technologies, Thousand Oaks, California, USA) mounted on an Olympus CKX53 microscope. Cells were normalised to 0.5 × 10^6^ cells per pellet in DPBS and intracellular iron content was measured colorimetrically at 590 nm using the Iron‐Assay‐Kit (Merck Sigma‐Aldrich; Cat.‐No. MAK472) according to the manufacturer's manual on a Spark multimode reader (Tecan).

### 2.7 GPX Activity

1 × 10^6^ cells were seeded in 100‐mm culture dishes (Sarstedt) and grown overnight. After 24 h, the medium was changed to −FBS, and after a further 24 h, cells were treated with the indicated substances. Cells were washed with DPBS (PAN‐Biotech) post‐treatment and scraped off with a rubber policeman. The steps for sample preparation and assessment of GPX activity using a Glutathione Peroxidase Assay Kit (Cayman Chemical; Cat.‐No. 703102) were conducted according to the manufacturer's instructions. GPX activity was measured colorimetrically at 340 nm in a Spark multimode reader (Tecan).

### 2.8 Western Blot

Cells were seeded in 100 mm dishes, grown for 24 h and starved for another 24 h in medium −FBS. Then, cells were washed with DBPS, harvested with trypsin‐EDTA, centrifuged (400 × *g*, 5 min), counted and stored as cell pellets at −20°C. For further processing, cell pellets were thawed, resuspended in DPBS to a concentration of 1 × 10^7^ cells per mL and lysed by sonication (10 pulses) with a Sonopuls HD70 (UW 70 ultrasound head; Bandelin electronic, Berlin, Germany). Samples were centrifuged (17,000 × *g*, 10 min at 4°C) and the supernatant was mixed with one volume of 2 × SDS (Thermo Fisher Scientific, Waltham, MA, USA), incubated for 5 min at 95°C and centrifuged (400 × *g*, 5 min at room temperature). Proteins were separated on gradient SDS gels (4%–20% Mini‐PROTEAN gels, Bio‐Rad, Hercules, CA, USA) for 90 min at 100 V, each slot containing the volume equivalent of 100,000 cells. Gels were then transferred to nitrocellulose membranes (Bio‐Rad) and blotted using a Trans‐Blot Turbo System (7 min, 25 V; Bio‐Rad). Membranes were incubated for 1 h with Blotting Grade Blocker (Bio‐Rad), followed by overnight incubation at 4°C with primary mouse monoclonal IgG_2b_κ anti‐GPX4‐antibody (Santa Cruz Biotechnology, Dallas, TX, USA; Cat.‐No. sc‐166570) diluted 1:500 and rabbit anti‐β‐actin‐antibody (Cell Signaling Technology, Danvers, MA, USA; Cat.‐No. 4970) diluted 1:2000. Blots were washed with TBS‐T and incubated for 1 h at room temperature with secondary goat anti‐mouse‐IgGκ BP‐horseradish peroxidase (HRP) antibody (Santa Cruz Biotechnology; Cat.‐No. sc‐516102) and HRP‐linked mouse anti‐rabbit‐IgG antibody (Cell Signaling Technology; Cat.‐No. 7074), respectively (both diluted 1:2000). Proteins were detected with Signal Fire ECL Reagent (Cell Signaling Technologies) and chemiluminescence was analysed using a ChemiDoc MP System (Bio‐Rad). β‐actin served as a loading control.

### 2.9 Statistical Analysis

GraphPad Prism 10 (GraphPad Software, Boston, MA, USA) was used for data visualisation and statistics. Graphs show data points of at least three individual experiments (biological replicates, *n* ≥ 3), unless otherwise specified. To provide a visual representation of the variability and precision of the estimated means, we include the 95% confidence intervals (CI; truncated at 0 where applicable) in all figures. The estimation of 95% CI assumes an underlying *t*‐distribution, which we justified by conducting the Shapiro–Wilk test. The results indicated that the normality assumption was reasonably met, supporting the validity of the confidence intervals presented, which was also evaluated visually with *Q*‐*Q* plots. Based on this assumption, statistical analyses were performed by repeated measures (RM) one‐way ANOVA with Geisser–Greenhouse correction for violations of sphericity, followed by Dunnett’s multiple comparisons post‐test or using paired *t*‐tests where appropriate. Results were considered statistically significant at *p* < 0.05.

## 3 Results

### 3.1 Viability Is Reduced in Chondrocyte Cell Lines C‐28/I2 and T/C‐28/A2 and in Primary Human Chondrocytes After Treatment With Ferroptosis Inducers

**FIGURE 1:**
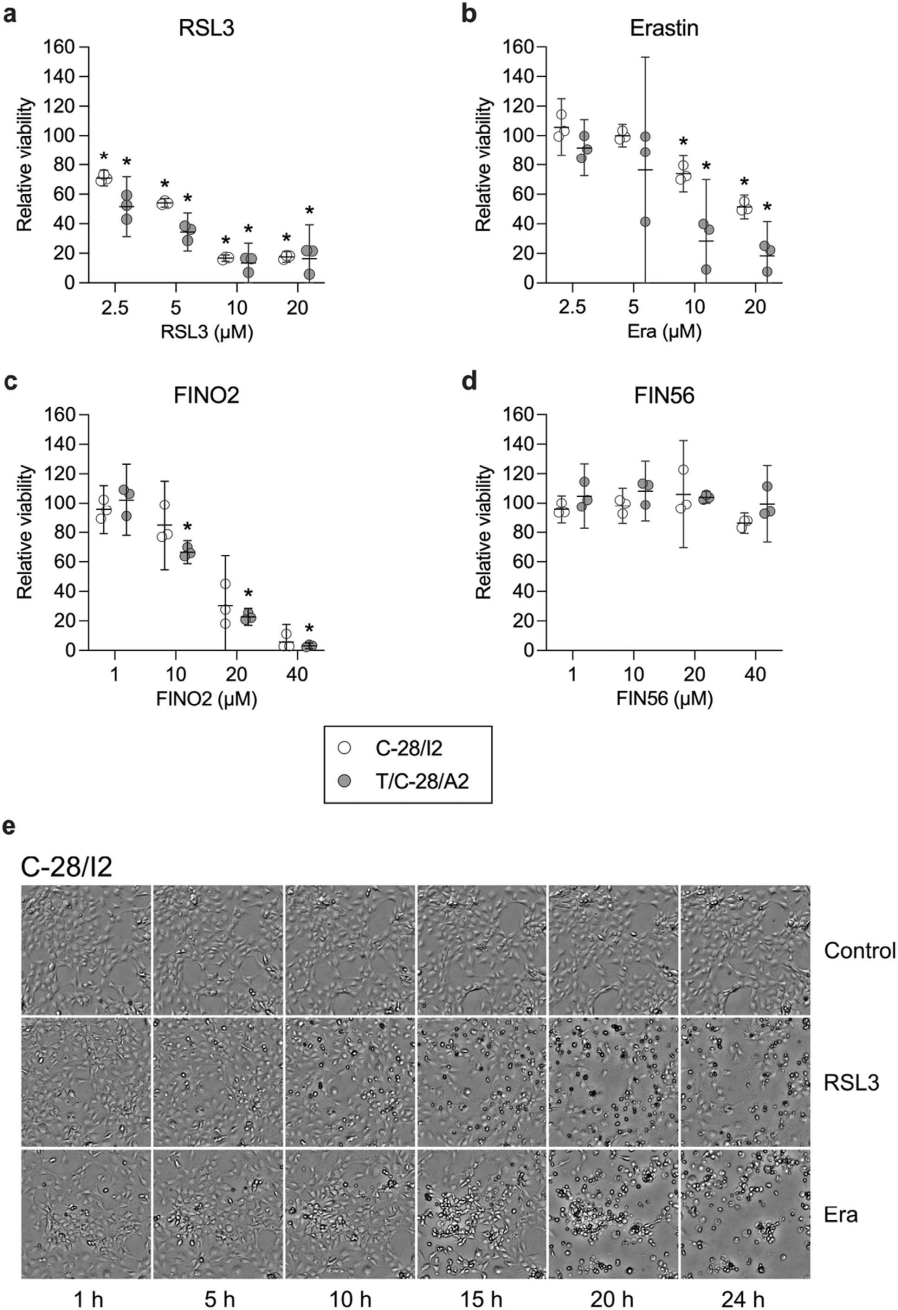
Viability is reduced in chondrocyte cell lines C‐28/I2 and T/C‐28/A2 after treatment with ferroptosis inducers. (a–d) Cell viability assessed by resazurin assays after 24 h of treatment with RSL3, erastin, FINO2 and FIN56 in % of untreated controls. Results of individual experiments (*n* = 3 for each substance) are shown as empty and grey symbols for C‐28/I2 and T/C‐28/A2 cells, respectively, with means (95% CI). RM one‐way ANOVA with Geisser–Greenhouse correction and Dunnett’s multiple comparisons post‐test. *p < 0.05 versus untreated controls. (e) Transmission microscopy images of C‐28/I2 cells taken every 5 h over 24 h in the absence (control) and presence of RSL3 (10 μM) or erastin (Era; 20 μM). 10× magnification.

We first examined the effects of FINs alone on C‐28/I2 and T/C‐28/A2 cells and primary chondrocytes and found a dose‐dependent reduction in cell viability after a 24‐h treatment with the respective substances (Figure 1a–d). RSL3 had the strongest effect on C‐28/I2 and T/C‐28/A2 cells, leading to nearly 30%–50% reduced viability already at the lowest concentration tested (2.5 μM). Likewise, erastin and FINO2 impaired cell viability but at higher concentrations (≥ 5 μM), whereas FIN56 did not markedly reduce viability. Generally, T/C‐28/A2 cells were more sensitive to the ferroptosis inducers than C‐28/I2 cells. Primary chondrocytes showed the highest sensitivity to the inducers and displayed ~50% reduced viability upon FIN56 treatment, which left the immortalised cell lines unaffected at the same and higher concentrations (Figure S1a).

The induction of cell death was also microscopically apparent. For C‐28/I2 cells treated with RSL3 (10 μM) or erastin (20 μM) for 24 h, Figure 1e shows a time‐dependent accumulation of rounded‐up (‘ballooning’) and dead cells over time, whereby the effects of RSL3 were apparent earlier than those of erastin. T/C‐28/A2 cells were morphologically affected in a similar way by RSL3 and erastin, again showing a higher sensitivity compared to C‐28/I2 cells. Especially after RSL3 treatment, changes in cell morphology and the appearance of dead cells were already obvious after 5 h (Figure S2e).

### 3.2 Iron Overload Enhances Ferroptosis Induction

**FIGURE 2:**
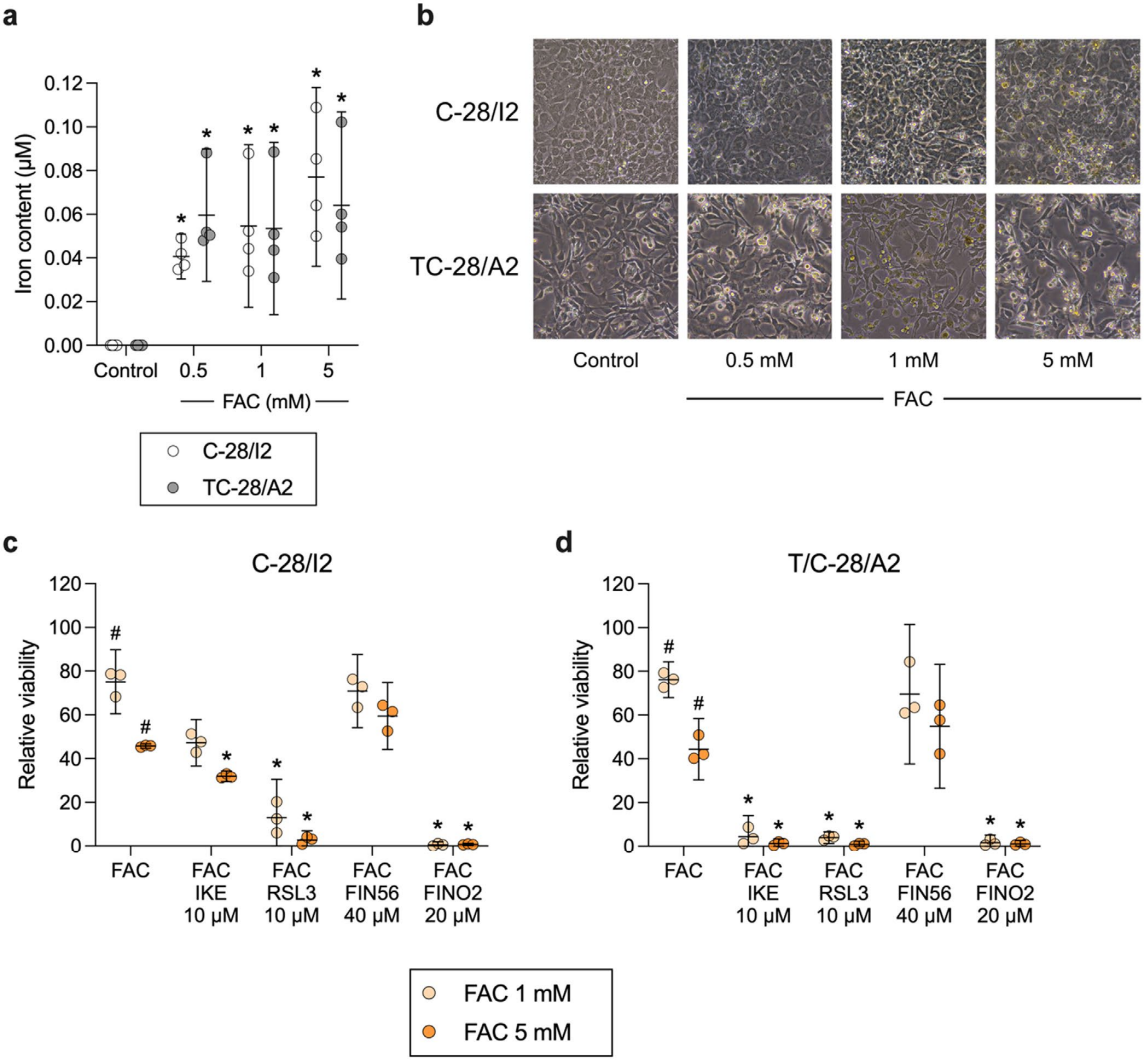
Iron overload enhances ferroptosis induction. (a) Iron content of C‐28/I2 (empty symbols) and TC‐28/A2 cells (grey symbols) cultured for 24 h in the absence (control) or presence of 0.5, 1 and 5 mM ferric ammonium citrate (FAC). Means (95% CI) of four individual experiments (*n* = 4). RM one‐way ANOVA with Geisser–Greenhouse correction and Dunnett’s multiple comparisons post‐test. *p < 0.05 versus Control. (b) Transmission images taken after 24 h under the same conditions as in (a). 20× magnification. Cell viability after 24 h treatment of (c) C‐28/I2 cells or (d) T/C‐28/A2 cells with 1 mM (light symbols) or 5 mM (dark symbols) FAC alone or in combination with IKE (10 μM), RSL3 (10 μM), FIN56 (40 μM) or FINO2 (20 μM). Results of individual resazurin experiments (*n* = 3 for each condition) in % of untreated controls and means (95% CI). RM one‐way ANOVA with Geisser–Greenhouse correction and Dunnett’s multiple comparisons post‐test, or paired *t*‐tests. *p < 0.05 versus FAC alone, ^#^ *p* < 0.05 versus untreated controls.

Next, we tested whether additional exposure to iron increases cell death. Therefore, cells were exposed to 1 and 5 mM ferric ammonium citrate (FAC) for 24 h. Lower concentrations from 100 to 500 μM have previously been shown to cause iron overload and reduced viability in chondrocytes [25, 26, 27]. We confirmed increased iron uptake after 24‐h exposure to 0.5, 1 and 5 mM FAC (Figure 2a), which was also microscopically visible. Higher FAC concentrations led to a higher number of rounded cells, indicating reduced viability (Figure 2b). Iron overloading caused a dose‐dependent reduction in cell viability by approximately 25% and 55% in C‐28/I2 and T/C‐28/A2 cells, respectively (Figure 2c,d). The effect of iron overload was enhanced by FINs. Combined treatment with FAC and RSL3 or IKE led to a stronger reduction of living cells than FAC alone. In C‐28/I2 cells, 31.94% and 2.71% of cells survived co‐treatment with 5 mM FAC plus IKE or RSL3 at 10 μM each, respectively (Figure 2c). These effects were again more prominent in T/C‐28/A2 cells (Figure 2d), whereupon co‐treatment only 1.24% and 0.86% of living cells were left, respectively. While FIN56 alone at the highest tested concentration of 40 μM left C‐28/I2 and T/C‐28/A2 cells unaffected (Figure 1d), additional iron overload reduced cell viability by 30%–50%. Combined treatment with FAC and FINO2 (20 μM), which alone already had strong effects, led to ultimate cell death in both cell lines.

### 3.3 The Ferroptosis Inhibitor Ferrostatin‐1 Partially Restores Cell Viability

**FIGURE 3:**
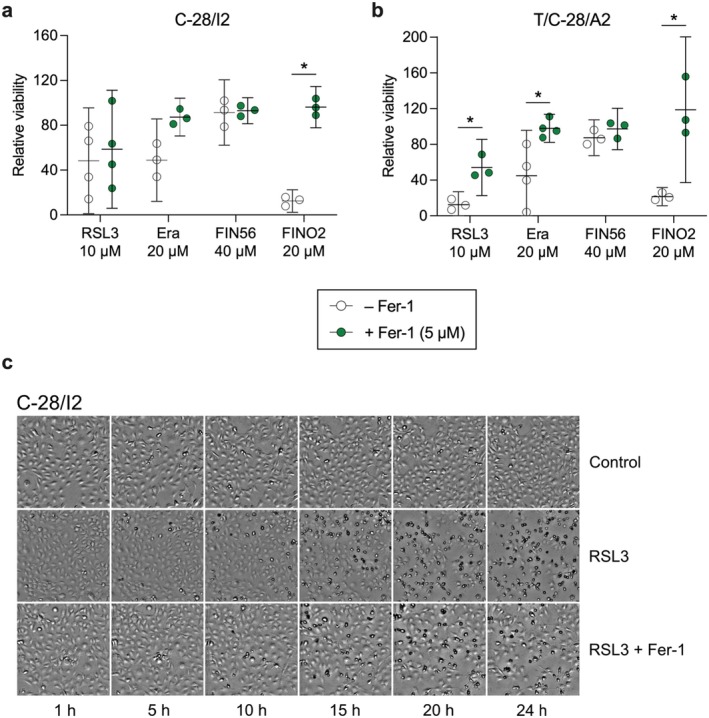
The ferroptosis inhibitor ferrostatin‐1 (Fer‐1) partially restores cell viability. (a, b) Cell viability of C‐28/I2 cells, T/C‐28/A2 cells after 24 h of treatment with RSL3, erastin, FIN56 and FINO2 alone (empty symbols) or in combination with Fer‐1 (5 μM; green symbols). Results of individual resazurin experiments (*n* = 3–4) in % of untreated controls and means (95% CI). Paired *t*‐tests. *p < 0.05. (c) Transmission microscopy images of C‐28/I2 cells taken every 5 h over 24 h in the absence (control) and presence of RSL3 (10 μM) and cells co‐treated with RSL3 and Fer‐1 (5 μM). 10× magnification.

To verify the ferroptotic effects of the inducers, we performed co‐treatment experiments with the ferroptosis inhibitor ferrostatin‐1 (Fer‐1). C‐28/I2 and T/C‐28/A2 cells were treated for 24 h with RSL3 (10 μM), erastin (20 μM), FIN56 (40 μM) and FINO2 (20 μM) alone, or in combination with 5 μM Fer‐1. In C‐28/I2 cells, Fer‐1 slightly ameliorated the effect of RSL3 in three out of four experiments, evident as increased viability in cells co‐treated with Fer‐1 (+10% relative viability on average) (Figure 3a). The effect of Fer‐1 was more prominent and consistent in T/C‐28/A2 cells, where relative viability rose from 12.5% to 54.2% (+41.7%) on average in the co‐treatment group (Figure 3b). In erastin‐treated cells, Fer‐1 co‐treatment comparably raised the viability in both cell lines (C‐28/I2: +38.4%, T/C‐28/A2: +53.0%). The strongest anti‐ferroptotic effect of Fer‐1 was apparent in FINO2‐treated cells, where the relative viability increased by 83.8% in C‐28/I2 and by 92.8% in T/C‐28/A2 cells, restoring the viability to the level of untreated control cells. Again, FIN56 had no discernible effect. Fer‐1 also inhibited ferroptotic cell death in primary chondrocytes, where RSL3 and IKE again exerted strong effects by reducing viability to < 2% compared to untreated cells. In combination with RSL3 or IKE, Fer‐1 restored viability to 16.1% and 41.7%, respectively (Figure S1b). We confirmed these results with a second assay based on luminescent detection of intra‐cellular ATP as a surrogate parameter for cell viability. Fer‐1 partially restored cell viability in RSL3 and FINO2‐treated C‐28/I2 and T/C‐28/A2 cells (Figure S2a,b) as well as in co‐treatment with FAC (Figure S2c,d). In C‐28/I2 cells, the inhibitory effect of Fer‐1 on RSL3‐induced cell death is also clearly visible microscopically (Figure 3c). For T/C‐28/A2 cells, a similar counteracting effect of Fer‐1 on erastin‐induced ferroptosis is shown in Figure S2e.

### 3.4 Ferroptotic Cell Death Is Accompanied by Minor Apoptotic and Necroptotic Effects

**FIGURE 4:**
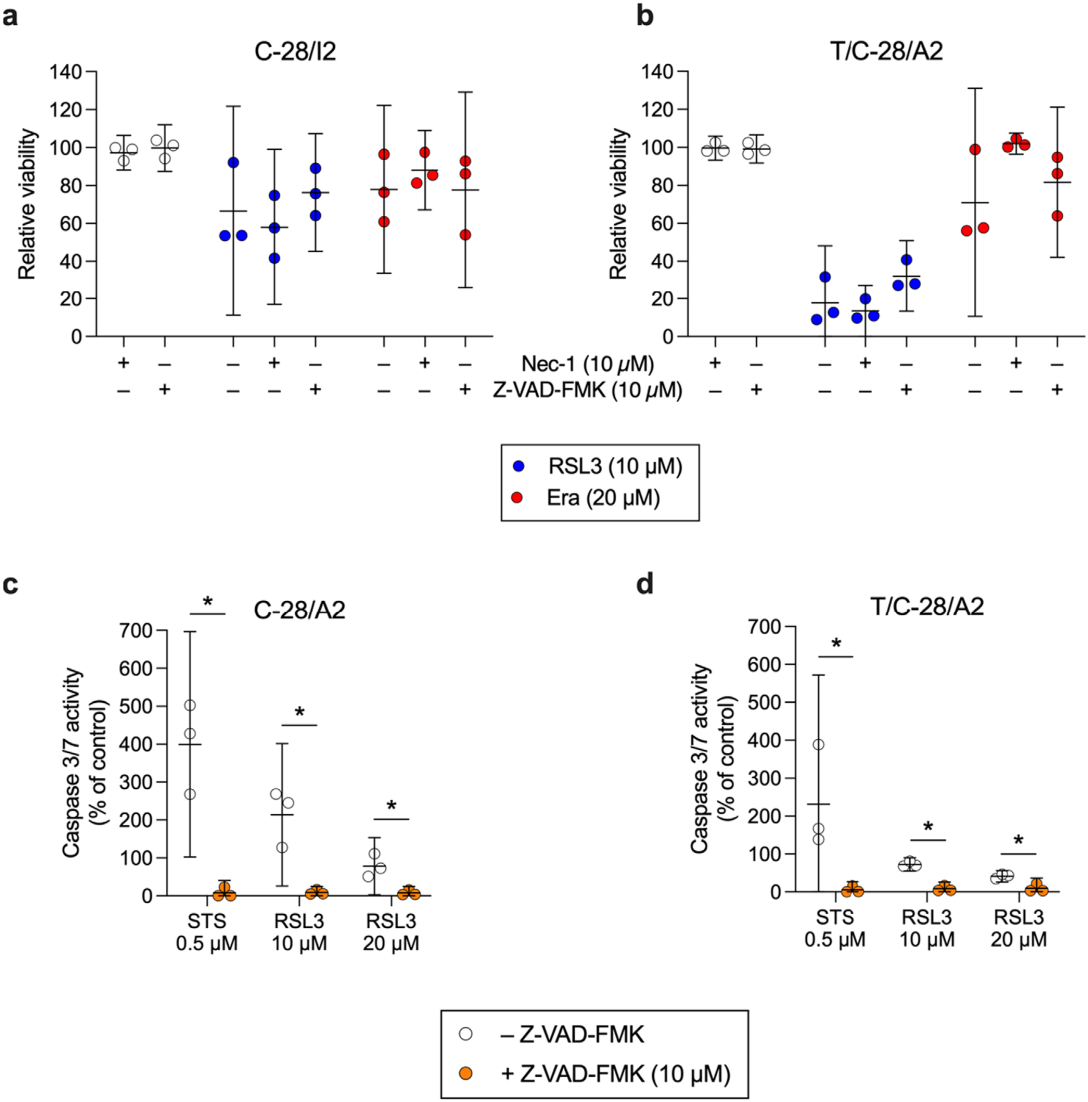
Ferroptotic cell death is accompanied by minor apoptotic and necroptotic effects. (a, b) Cell viability of C‐28/I2 and T/C‐28/A2 cells treated for 24 h with the necroptosis inhibitor necrostatin‐1 (Nec‐1; 10 μM) or the apoptosis inhibitor Z‐VAD‐FMK (10 μM) alone (empty symbols), or in combination with RSL3 (10 μM; blue symbols) or erastin (Era; 20 μM; red symbols). Results of individual resazurin measurements (*n* = 3) in % of untreated controls and means (95% CI). (c, d) Caspase 3/7 activity in C‐28/I2 and T/C‐28/A2 treated with staurosporine (STS; 0.5 μM; positive control) or RSL3 (10 and 20 μM) in the absence (empty symbols) and presence (orange symbols) of Z‐VAD‐FMK (10 μM). Results of individual Caspase‐Glo 3/7 assays (Promega) in % of untreated controls (*n* = 3–4) and means (95% CI). Paired *t*‐tests. *p < 0.05.

To test for a possible contribution of apoptotic and necroptotic processes, we co‐treated chondrocytes with RSL3 (10 μM) or erastin (20 μM), along with Z‐VAD‐FMK (10 μM) or necrostatin‐1 (Nec‐1; 10 μM). Z‐VAD‐FMK prevents apoptosis by inhibiting caspases, whereas Nec‐1 inhibits receptor interacting serine/threonine kinase1 (RIP1 kinase) and necroptotic cell death. In both cell lines, the treatment with RSL3 plus Nec‐1 for 24 h slightly enhanced the cytotoxic effect (C‐28/I2: −8.4%, T/C‐28/A2: −4.3% relative viability compared to RSL3 alone) (Figure 4a,b). Since 10 μM Nec‐1 alone had no effect, this could be caused by the combination of the two substances. In contrast, when applied together with RSL3, Z‐VAD‐FMK increased the viability by 9.9% in C‐28/I2 and by 14.3% in T/C‐28/A2 cells, indicating an involvement of apoptotic mechanisms. The co‐treatment of erastin with the two inhibitors revealed a different pattern. In both cell lines, treatment with erastin in combination with Nec‐1 increased cell viability (C‐28/I2: +10.2%, T/C‐28/A2: +31.1%), suggesting an involvement of necrosis besides ferroptosis. The combination with Z‐VAD‐FMK did not change the effects of erastin in C‐28/I2 cells, but in T/C‐28/A2 cells, the viability was raised by 10.8% when both substances were applied (Figure 4b).

By quantifying caspase 3/7 activity, we found that compared to the apoptosis inducer staurosporine (STS), RSL3 elicited less than 50% caspase activity in both cell lines, indicating a weaker apoptotic effect. Z‐VAD‐FMK inhibited caspase activation in both RSL3 and staurosporine‐treated cells, which confirms the involvement of apoptotic effects in cells treated with RSL3 (Figure 4c,d).

### 3.5 CBD Partially Inhibits Ferroptotic Cell Death in Human Articular Chondrocytes

**FIGURE 5:**
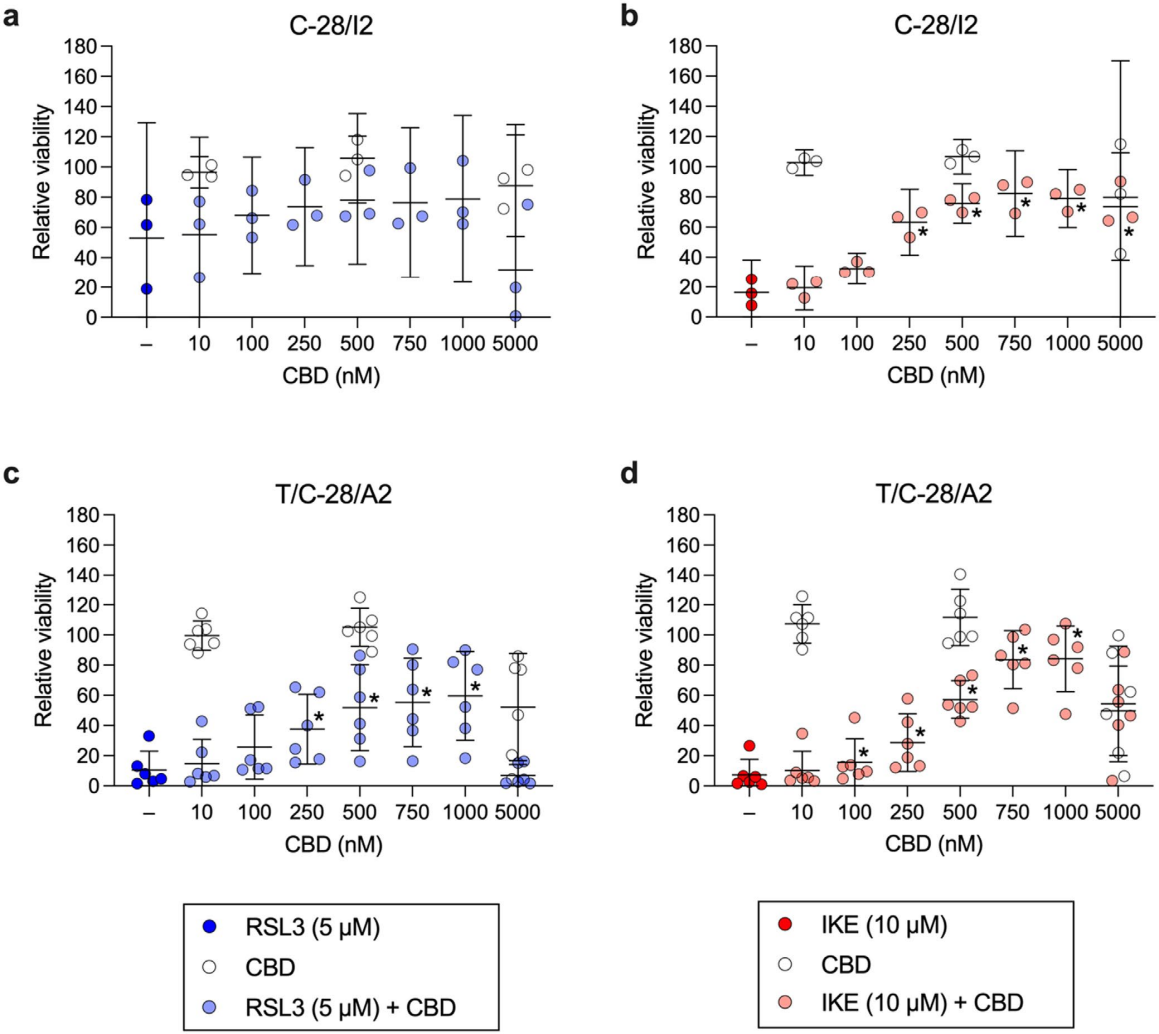
CBD partially inhibits ferroptotic cell death in human articular chondrocytes. Cell viability of C‐28/I2 cells (a, b), T/C‐28/A2 cells (c, d) after 24 h treatment with RSL3 (5 μM; blue symbols) or IKE (10 μM; red symbols) alone or in combination with cannabidiol (CBD) at concentrations ranging from 10 to 5000 nM. Results from cells treated with 10, 500 and 5000 nM CBD alone are depicted as empty symbols. Results of individual resazurin experiments in % of untreated controls (*n* = 3 and 6 for C‐28/I2 and T/C‐28/A2 cells, respectively) and means (95% CI). RM one‐way ANOVA with Geisser–Greenhouse correction and Dunnett’s multiple comparisons post‐test. *p < 0.05 RSL3 or IKE plus CBD versus RSL3 or IKE alone.

Next, we focused on the effects of CBD on chondrocyte viability under ferroptotic conditions. Rising concentrations of CBD from 10 to 1000 nM dose‐dependently ameliorated the effect of 5 μM RSL3 in C‐28/I2 and T/C‐28/A2 cells (+25.9% and +49.1% relative viability at 1000 nM CBD, respectively), while CBD alone up to 500 nM had no effect (Figure 5a,c). At the highest tested concentration of 5000 nM, CBD per se reduced cell viability, indicating a cytotoxic effect of CBD at higher concentrations, as previously described [22]. In primary chondrocytes, CBD had the most consistent and pronounced cytoprotective effect: viability rose by more than 76% from RSL3‐only treated cells to cells co‐treated with 1000 nM CBD. Again, at the highest tested concentration, CBD caused cell death when applied alone and even more when combined with RSL3 (Figure S1c). CBD also mitigated cell viability in IKE‐treated cells: in C‐28/I2 and T/C‐28/A2 cells, the percentage of viable cells strongly rose from 16.2% and 7.4% under 10 μM IKE alone to 79.0% and 84.2% in cells co‐treated with 1000 nM CBD, respectively. In both cell lines, CBD at 5000 nM again diminished viability (Figure 5b,d). In primary chondrocytes, co‐treatment with CBD up to 1000 nM gave rise to a similar rescue pattern. Notably, the cytotoxic effect of 5000 nM CBD was stronger and more consistent than in the two cell lines (Figure S1d). CBD also reduced the ferroptotic effects of FINO2 in C‐28/I2 and T/C‐28/A2 cells, but the effects were less pronounced compared to RSL3 and IKE; CBD co‐treatment improved the viability by < 20% in both cell lines (data not shown). Luminescence‐based quantification of intra‐cellular ATP levels also shows a dose‐dependent increase in viability in C‐28/I2 and T/C‐28/A2 cells co‐treated with CBD and RSL3 or IKE, confirming the results from resazurin assays (Figure S3a–d). The rescuing effect was also discernible in C‐28/I2 cells treated with RSL3 plus FAC (Figure S3e).

### 3.6 CBD Partially Restores GPX4 Activity in Ferroptotic Human Articular Chondrocytes

**FIGURE 6:**
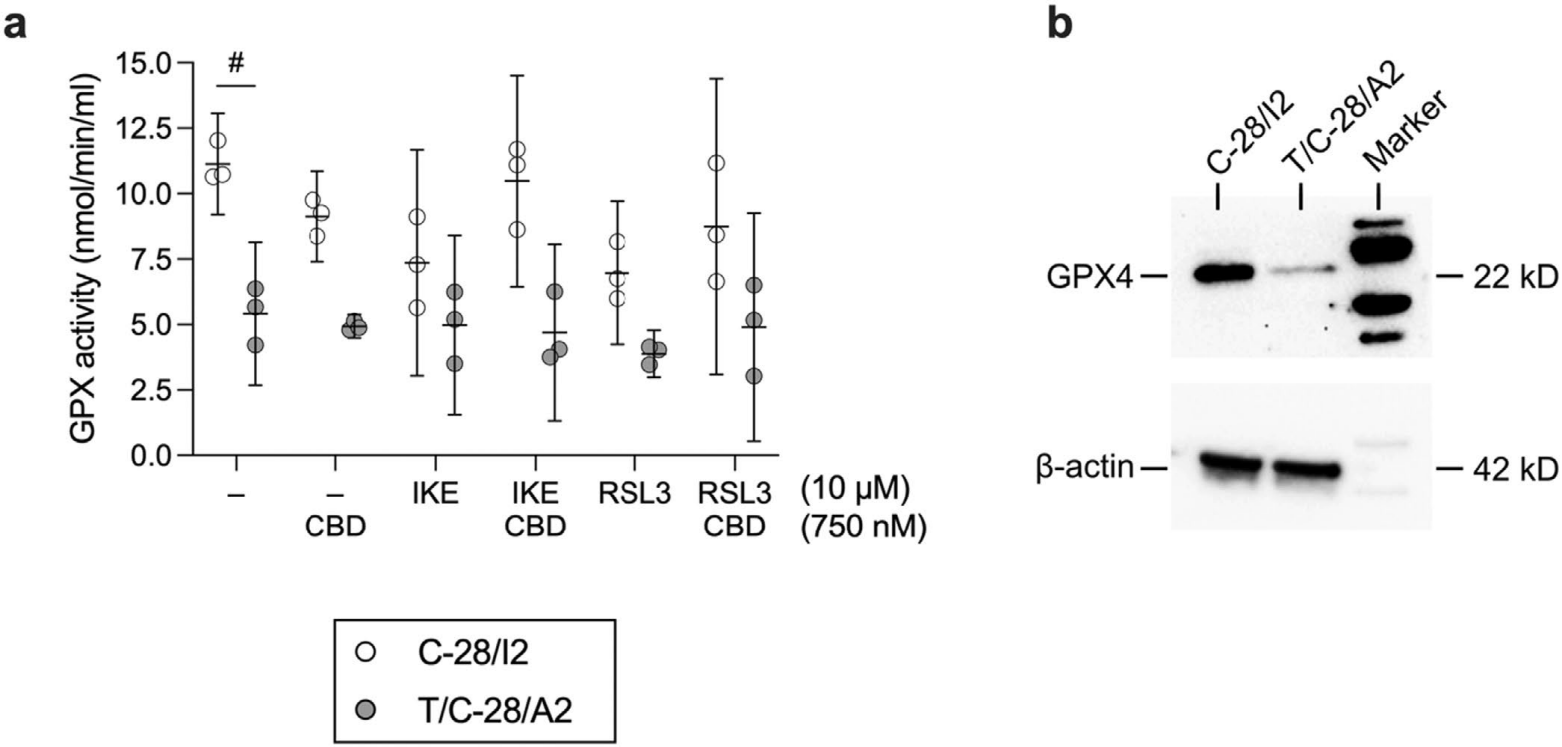
CBD partially restores GPX4 activity in ferroptotic human articular chondrocytes. (a) GPX activity in untreated C‐28/I2 and T/C‐28/A2 cells (−), in cells treated for 24 h with CBD (750 nM), IKE (10 μM) or RSL3 (10 μM) alone and cells co‐treated with CBD and ferroptosis inducers. GPX activity is given in nmol/min/mL for individual experiments (*n* = 3 for each cell line) and as means (95% CI). Paired *t*‐test. ^#^ *p* < 0.05. (b) Western blot analysis of GPX4 expression in C‐28/I2 and T/C‐28/A2 cells. β‐Actin served as loading control.

GPX4 is the main enzyme responsible for the reduction of lipid peroxides, and CBD is known to influence the oxidative state in different cell types. We investigated the effect of CBD alone and in combination with RSL3 and IKE on GPX activity in C‐28/I2 and T/C‐28/A2 cells. CBD alone (750 nM) reduced GPX activity in both cell lines, but cell type‐dependently restored GPX activity when co‐applied with FINs. Specifically, in C‐28/I2 cells, IKE and RSL3 (10 μM each) diminished GPX activity by 34.0% and 37.4% on average within 24 h. The effect of IKE was almost fully prevented by CBD (+28.1% activity versus IKE alone), while the RSL3 effect was recovered by ~16% (Figure 6a). In untreated T/C‐28/A2 cells, GPX activity was approximately 50% of the activity in C‐28/I2 cells, which is well reflected by the lower GPX4 protein expression in these cells (Figure 6b). From this lower level of GPX activity, IKE alone did only slightly reduce activity by 8.1%. With −28.4% activity versus untreated cells, the effect of RSL3 was more pronounced, suggesting that within 24 h, direct inhibition of GPX4 is less tolerated by these cells than system x_c_^−^ transport inhibition by IKE. The RSL3 effect was fully prevented by CBD in two out of three experiments (Figure 6a).

## 4 Discussion

In this study, we investigated the impact of CBD in human articular chondrocytes in the context of ferroptosis. Chondrocytes produce and maintain the extracellular matrix (ECM) in hyaline cartilage. Injury, mechanical overload, genetic‐ and biochemical factors can lead to matrix degradation and chondrocyte cell death. Ferroptosis has been linked to cartilage degeneration and ECM degradation in chondrocyte cell models and in vivo studies (see Cao et al. [20] for a review).

In C‐28/I2 cells, T/C‐28/A2 cells and primary chondrocytes, we observed a dose‐dependent decline in cell viability upon the application of the FINs erastin or its analogue IKE, RSL3 and FINO2. This indicates that cysteine depletion by inhibition of antiport system x_c_^−^ (erastin/IKE), direct inhibition of GPX4 (RSL3) and promotion of iron oxidation, and indirect inhibition of GPX4 (FINO2) elicited ferroptotic cell death. Treatment with FIN56, which promotes GPX4 protein degradation, had only weak effects on cell viability within 24 h, suggesting a slower effect or a potential compensatory ability of the cells towards FIN56. The two cell lines displayed remarkable differences in their compound sensitivity, which was also microscopically apparent; T/C‐28/A2 cells were generally more prone to ferroptosis, suggesting cell type‐dependent anti‐ferroptotic capacity, as known from other cell types [28]. Compared to the immortalised cell lines, primary chondrocytes showed the highest sensitivity to the tested compounds overall, with almost all cells dead after 24‐h treatment with the highest concentration of IKE, RSL3 or FINO2. Primary chondrocytes were also more sensitive to FIN56, suggesting a lower level cellular antioxidant defence system.

Induction of ferroptosis and disruption of cartilage metabolism by iron overload upon exposure to ferric ammonium citrate (FAC) has been described in chondrocyte cell lines and primary chondrocytes [25, 26, 27, 29, 30, 31] and we confirm these results. FAC reduced cell viability, and co‐treatment with FINs markedly enhanced that effect. On the other hand, co‐application of the radical scavenger ferrostatin‐1 (Fer‐1) with FINs partially restored cell viability in both cell lines and in primary chondrocytes. While our experiments clearly show the induction of ferroptosis, simultaneous activation of necroptotic or apoptotic pathways is likely. This is consistent with studies showing that although ferroptosis is morphologically, genetically and biochemically distinct, there may be crosstalk or mutual activation of apoptosis and other forms of non‐apoptotic cell death [32, 33]. The elevated caspase 3/7 activity we observed in cells treated with RSL3 indicates a pro‐apoptotic effect in addition to its well‐known ferroptotic effect of the compound. This finding aligns with recent work by Liu et al. [34], who reported increased apoptosis in myelodysplastic syndrome cells following RSL3 treatment, accompanied by elevated levels of cleaved caspase 3. Notably, in our experiments, caspase 3/7 activation was reduced at the higher RSL3 concentration of 20 μM compared to 10 μM, suggesting a potential shift towards caspase‐independent cell death at higher concentrations.

Inhibition of ferroptosis by specific inhibitors, iron chelators or unspecific antioxidants alleviates OA‐cartilage damage and ECM degradation by multiple mechanisms [25, 29, 31, 35, 36, 37, 38]. CBD affects redox balance through both pro‐oxidative and anti‐oxidative effects in a cell type‐specific, dose‐ and context‐dependent manner. Antioxidant effects can be explained by its ability to directly reduce oxidant levels and to prevent the formation of superoxide radicals. CBD also reduces ROS production by chelation of transition metal ions and indirectly alters the levels and the activities of antioxidants [21, 39]. Few studies have investigated the effects of CBD or the endocannabinoid (EC) system in the context of ferroptosis. Li et al. described an anti‐ferroptotic effect of CBD at 0.08–10 μM in human skin keratinocytes [40] and Huang et al. demonstrated a cytoprotective effect of CBD at 10 μM in radiation‐induced enteritis through GPX4 transactivation [41], indicating enhanced ferroptosis resistance under CBD. On the other hand, a pro‐ferroptotic effect of CBD at 10–50 μM via ERK activation and ROS accumulation was found by Kim et al. in glioblastoma cells [42]. Sensitisation of breast cancer cells to ferroptosis by inhibition of the CB1‐receptor points at a potential involvement of the EC system in ferroptosis [43]. In chondrocytes, cytoprotective [44, 45, 46] as well as pro‐apoptotic [22, 47] effects of cannabinoids have been observed. We show that CBD, co‐applied at concentrations from 10 nM to 1 μM with FINs, exerts a cytoprotective effect in C‐28/I2 and T/C‐28/A2 cells and in primary chondrocytes with an efficacy in the mid‐nanomolar range comparable to the anti‐ferroptotic effect of 5 μM Fer‐1.

At 0.75 μM CBD alone reduced GPX activity in both C‐28/I2 and T/C‐28/A2 cells, but when combined with the FINs IKE or RSL3, it restored GPX activity to varying degrees depending on the cell type. At the same concentration, CBD improved cell viability in the presence of RSL3 or IKE in both cell lines, suggesting that the effect of CBD on GPX activity is not the sole mechanism counteracting RSL3‐ and IKE‐induced cell death. For example, IKE at 10 μM drastically reduced the viability of T/C‐28/A2 cells without significantly affecting GPX activity, but CBD at 750 nM effectively counteracted IKE‐induced cell death in these cells, while GPX activity remained largely unchanged. The mechanism by which CBD affects GPX4 activity remains to be elucidated, including whether it involves direct or indirect GPX activation and/or increased GPX expression.

Our study is the first time to identify an anti‐ferroptotic effect of cannabidiol (CBD) in human articular chondrocytes. Current focus on disturbed iron homeostasis in ageing‐related diseases highlights a role of ferroptosis in the pathogenesis of OA, the most prevalent joint disease affecting millions of people worldwide. Given the growing recognition of ferroptosis in OA pathology, the here described anti‐ferroptotic effect of CBD provides a basis for future research into CBD‐based approaches aimed at preserving chondrocyte viability and mitigating ferroptosis‐driven cartilage degradation, with potential for pharmacological interventions in OA treatment.

## Author Contributions

**A. Wipplinger:** data curation (supporting), investigation (lead), methodology (equal), project administration (supporting), validation (supporting), visualization (supporting), writing – original draft (supporting), writing – review and editing (supporting). **D. Bekric:** conceptualization (supporting), data curation (supporting), formal analysis (supporting), methodology (supporting), validation (supporting), writing – original draft (supporting), writing – review and editing (supporting). **C. Ablinger:** investigation (supporting), methodology (supporting), writing – original draft (supporting). **M. Kittl:** data curation (supporting), formal analysis (supporting), investigation (supporting), methodology (supporting), validation (supporting), writing – original draft (supporting). **C. Mayr:** conceptualization (supporting), methodology (supporting), validation (equal), writing – original draft (supporting). **M. Ritter:** conceptualization (supporting), formal analysis (supporting), resources (lead), supervision (supporting), validation (supporting), writing – original draft (supporting), writing – review and editing (supporting). **M. Winklmayr:** conceptualization (equal), data curation (equal), formal analysis (equal), funding acquisition (lead), investigation (supporting), methodology (equal), supervision (supporting), validation (equal), visualization (equal), writing – original draft (equal), writing – review and editing (equal). **M. Jakab:** conceptualization (equal), data curation (equal), formal analysis (equal), funding acquisition (supporting), investigation (supporting), methodology (lead), project administration (supporting), supervision (lead), validation (lead), visualization (lead), writing – original draft (lead), writing – review and editing (lead).

## Conflicts of Interest

The authors declare no conflicts of interest.

## Supporting information


**Figure S1.** (a) Viability is reduced in primary human chondrocytes after treatment with ferroptosis inducers. Cell viability assessed by resazurin assays after 24 h of treatment with RSL3 (10 μM), IKE (20 μM), FINO2 (20 μM) and FIN56 (20 μM) in % of untreated controls. Results of three individual experiments (*n* = 3) for each substance are shown along with means (95% CI). Paired *t*‐tests. *p < 0.05 versus untreated controls. (b) The ferroptosis inhibitor ferrostatin‐1 (Fer‐1) partially restores cell viability in primary chondrocytes. Cell viability after 24 h of treatment with RSL3 (10 μM) or IKE (10 μM) alone (empty symbols) or in combination with Fer‐1 (5 μM; green symbols). Results of individual resazurin experiments (*n* = 3) in % of untreated controls and means (95% CI). Paired *t*‐tests. *p < 0.05. (c, d) CBD partially inhibits ferroptotic cell death in primary chondrocytes. Cell viability after 24‐h treatment with RSL3 (5 μM; blue symbols) or IKE (10 μM; red symbols) alone or in combination with cannabidiol (CBD) at concentrations ranging from 10 to 5000 nM. Results from cells treated with 10, 500 and 5000 nM CBD alone are depicted as empty symbols. Results of individual resazurin experiments in % of untreated controls (*n* = 3) and means (95% CI). RM one‐way ANOVA with Geisser–Greenhouse correction and Dunnett’s multiple comparisons post‐test. *p < 0.05 RSL3 or IKE plus CBD versus RSL3 or IKE alone.

**Figure S2.** The ferroptosis inhibitor ferrostatin‐1 (Fer‐1) partially restores cell viability. (a, b) Cell viability of C‐28/I2 and T/C‐28/A2 cells treated with RSL3 or FINO2 alone (empty symbols) and in combination with 5 μM Fer‐1 (green symbols). (c, d) Cell viability of C‐28/I2 and T/C‐28/A2 cells co‐treated with RSL3 or FINO2 and FAC (10 μM) (empty symbols) and in combination with 5 μM Fer‐1 (green symbols). Results of individual CellTiter‐Glo (Promega)‐measurements in % of untreated controls (*n* = 3–4 except for RSL3 plus FAC in c, where *n* = 2) and means (95% CI). Paired *t*‐tests. *p < 0.05. (e) Transmission microscopy images of T/C‐28/A2 cells taken every 5 h over 24 h in the absence (control) and presence of RSL3 (10 μM) or erastin (Era; 20 μM) and of cell co‐treated with Era and Fer‐1 (5 μM). 10× magnification.

**Figure S3.** CBD partially inhibits ferroptotic cell death in human articular chondrocytes. Cell viability of C‐28/I2 cells (a, b) and T/C‐28/A2 cells (c, d) after 24‐h treatment with RSL3 (10 μM; blue symbols) or IKE (10 μM; red symbols) alone or in combination with cannabidiol (CBD) at concentrations ranging from 10 to 750 nM. Results from cells treated with 750 nM CBD alone are shown as empty symbols. (e) Cell viability of C‐28/I2 cells after 24‐h treatment with RSL3 (10 μM) plus ferric ammonium citrate (FAC; 10 mM) in combination with 500 and 750 nM CBD. Results of individual CellTiter‐Glo assays in % of untreated controls and means (95% CI) (*n* = 3 for all conditions and both cell lines). RM one‐way ANOVA with Geisser–Greenhouse correction and Dunnett’s multiple comparisons post‐test. *p < 0.05 IKE plus CBD versus IKE alone.

## Data Availability

Data openly available in a public repository that issues datasets with DOIs.
