# Supplementary figures and images for: Cannabidiol Is a Potential Inhibitor of Ferroptosis in Human Articular Chondrocytes
Source: J Cell Mol Med. 2025 Jun 27;29(13):e70592. doi: 10.1111/jcmm.70592 (PMC12203570; doi:10.1111/jcmm.70592)

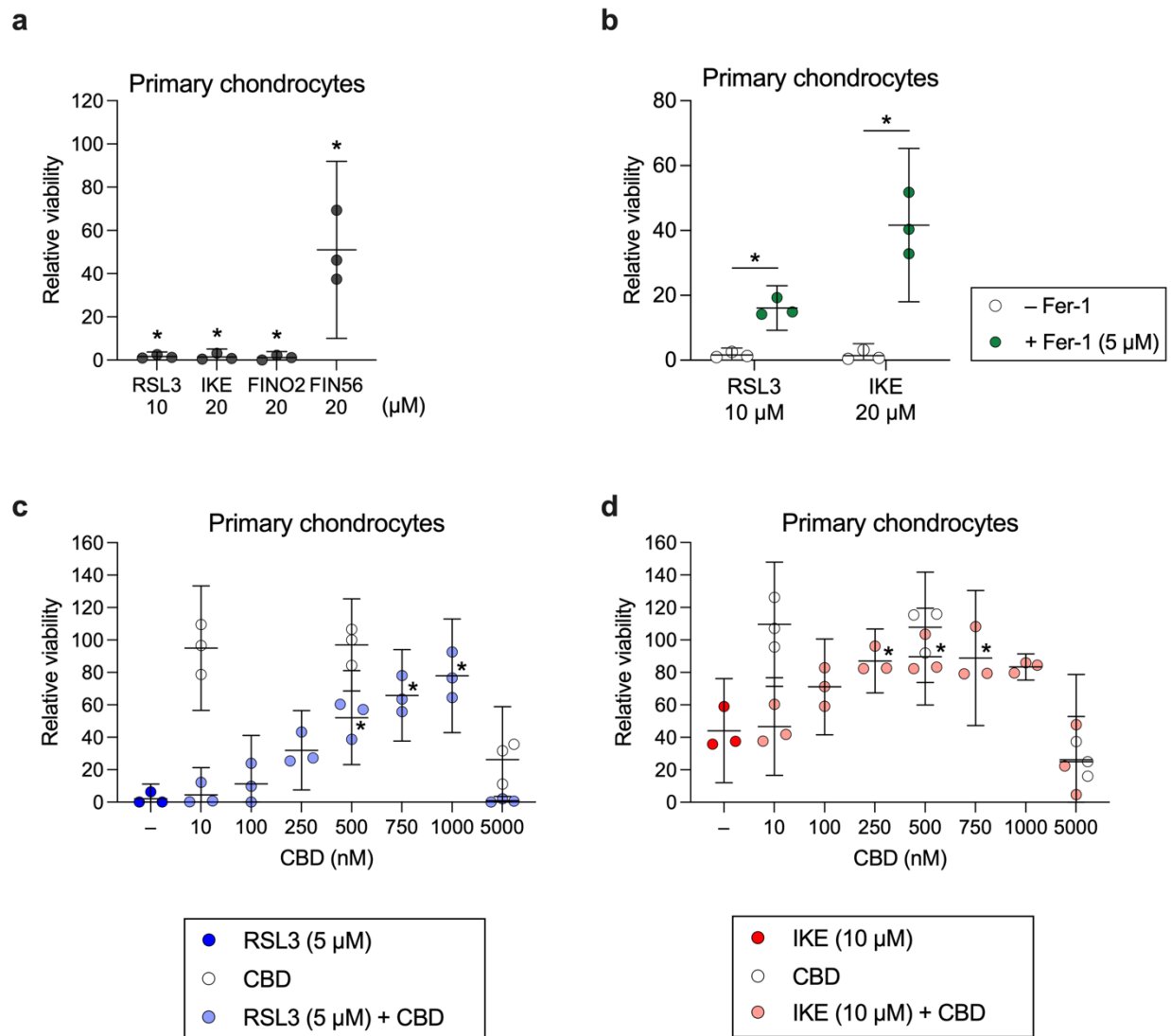

**Figure S1**

Supplement: Supplementary file 1 — Figure S1. (a) Viability is reduced in primary human chondrocytes after treatment with ferroptosis inducers. Cell viability assessed by resazurin assays after 24 h of treatment with RSL3 (10 μM), IKE (20 μM), FINO2 (20 μM) and FIN56 (20 μM) in % of untreated controls. Results of three individual experiments (n = 3) for each substance are shown along with means (95% CI). Paired t‐tests. *p < 0.05 versus untreated controls. (b) The ferroptosis inhibitor ferrostatin‐1 (Fer‐1) partially restores cell viability in primary chondrocytes. Cell viability after 24 h of treatment with RSL3 (10 μM) or IKE (10 μM) alone (empty symbols) or in combination with Fer‐1 (5 μM; green symbols). Results of individual resazurin experiments (n = 3) in % of untreated controls and means (95% CI). Paired t‐tests. *p < 0.05. (c, d) CBD partially inhibits ferroptotic cell death in primary chondrocytes. Cell viability after 24‐h treatment with RSL3 (5 μM; blue symbols) or IKE (10 μM; red symbols) alone or in combination with cannabidiol (CBD) at concentrations ranging from 10 to 5000 nM. Results from cells treated with 10, 500 and 5000 nM CBD alone are depicted as empty symbols. Results of individual resazurin experiments in % of untreated controls (n = 3) and means (95% CI). RM one‐way ANOVA with Geisser–Greenhouse correction and Dunnett’s multiple comparisons post‐test. *p < 0.05 RSL3 or IKE plus CBD versus RSL3 or IKE alone. [file JCMM-29-e70592-s001.pdf]

**a-d**  
CellTiter-Glo® Luminescent Cell Viability Assay (Promega)

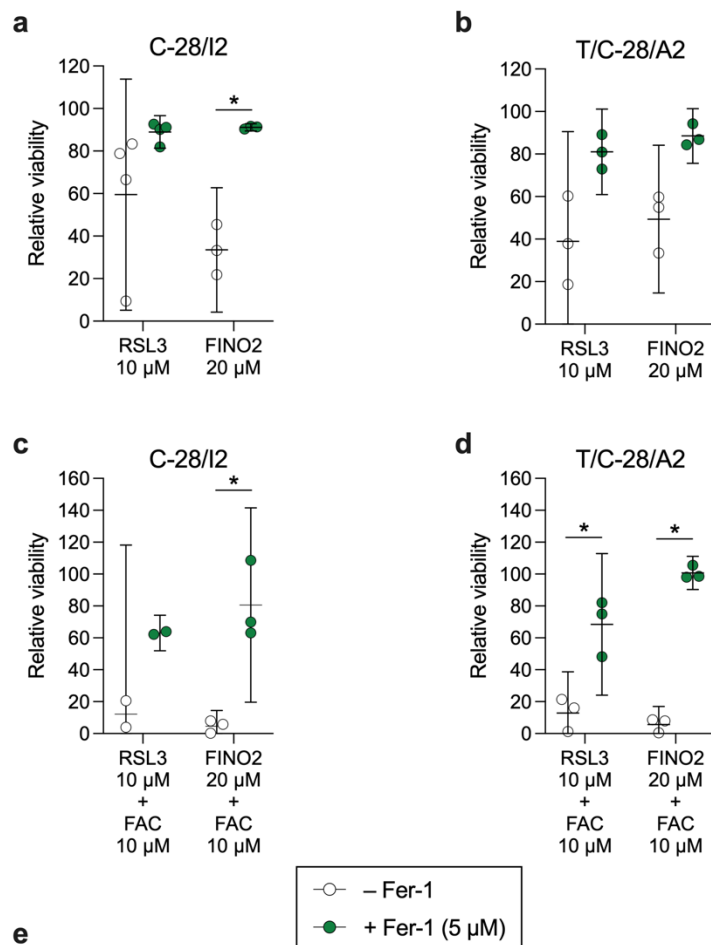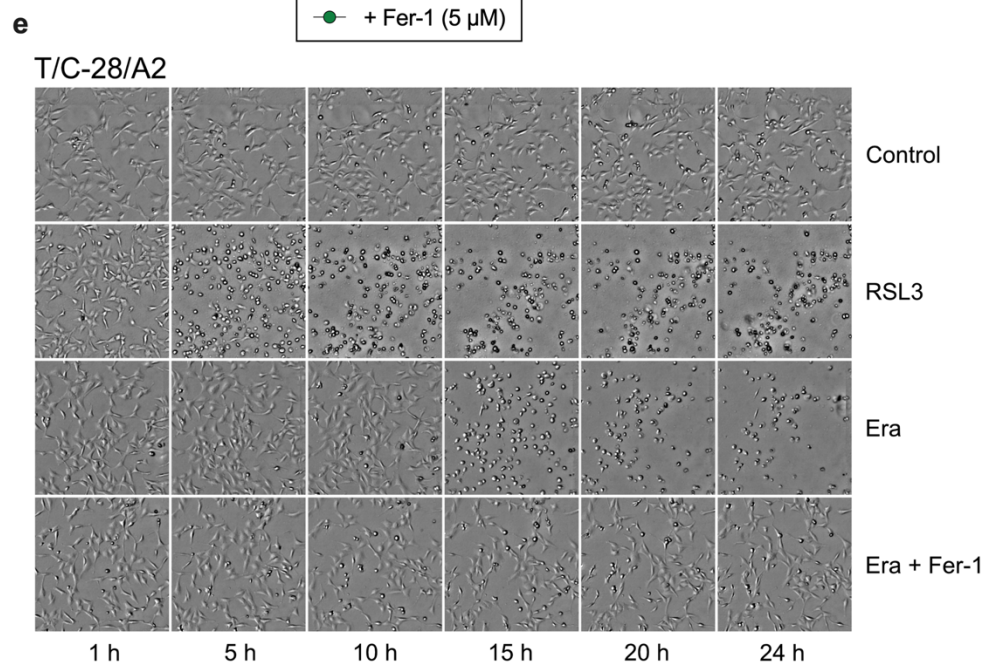

**Figure S2**

Supplement: Supplementary file 2 — Figure S2. The ferroptosis inhibitor ferrostatin‐1 (Fer‐1) partially restores cell viability. (a, b) Cell viability of C‐28/I2 and T/C‐28/A2 cells treated with RSL3 or FINO2 alone (empty symbols) and in combination with 5 μM Fer‐1 (green symbols). (c, d) Cell viability of C‐28/I2 and T/C‐28/A2 cells co‐treated with RSL3 or FINO2 and FAC (10 μM) (empty symbols) and in combination with 5 μM Fer‐1 (green symbols). Results of individual CellTiter‐Glo (Promega)‐measurements in % of untreated controls (n = 3–4 except for RSL3 plus FAC in c, where n = 2) and means (95% CI). Paired t‐tests. *p < 0.05. (e) Transmission microscopy images of T/C‐28/A2 cells taken every 5 h over 24 h in the absence (control) and presence of RSL3 (10 μM) or erastin (Era; 20 μM) and of cell co‐treated with Era and Fer‐1 (5 μM). 10× magnification. [file JCMM-29-e70592-s002.pdf]

CellTiter-Glo® Luminescent Cell Viability Assay (Promega)

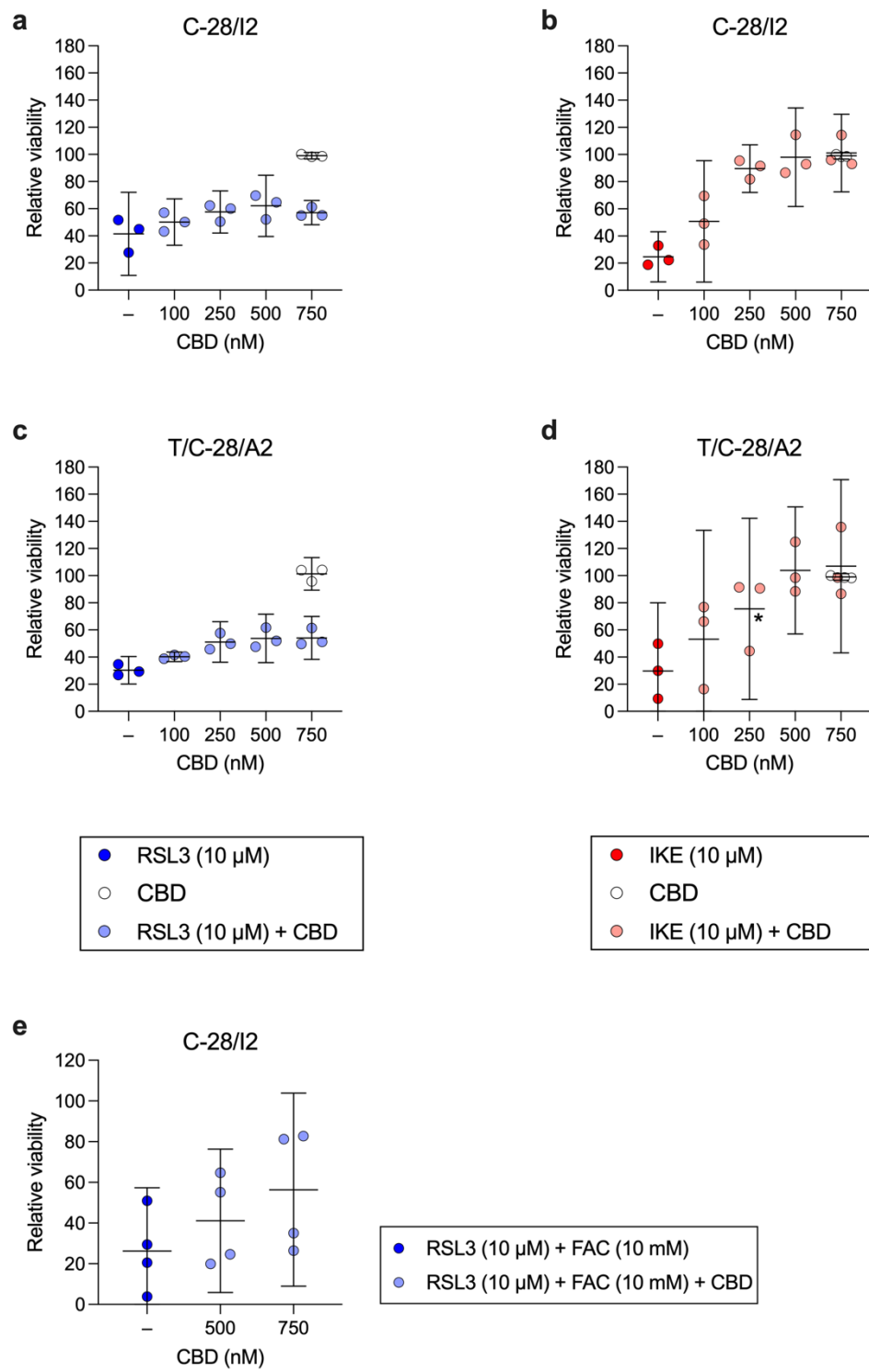

Figure S3

Supplement: Supplementary file 3 — Figure S3. CBD partially inhibits ferroptotic cell death in human articular chondrocytes. Cell viability of C‐28/I2 cells (a, b) and T/C‐28/A2 cells (c, d) after 24‐h treatment with RSL3 (10 μM; blue symbols) or IKE (10 μM; red symbols) alone or in combination with cannabidiol (CBD) at concentrations ranging from 10 to 750 nM. Results from cells treated with 750 nM CBD alone are shown as empty symbols. (e) Cell viability of C‐28/I2 cells after 24‐h treatment with RSL3 (10 μM) plus ferric ammonium citrate (FAC; 10 mM) in combination with 500 and 750 nM CBD. Results of individual CellTiter‐Glo assays in % of untreated controls and means (95% CI) (n = 3 for all conditions and both cell lines). RM one‐way ANOVA with Geisser–Greenhouse correction and Dunnett’s multiple comparisons post‐test. *p < 0.05 IKE plus CBD versus IKE alone. [file JCMM-29-e70592-s003.pdf]
